# Changes in the SARS-CoV-2 cellular receptor ACE2 levels in cardiovascular patients: a potential biomarker for the stratification of COVID-19 patients

**DOI:** 10.1007/s11357-021-00467-2

**Published:** 2021-10-21

**Authors:** Miklós Fagyas, Viktor Bánhegyi, Katalin Úri, Attila Enyedi, Erzsébet Lizanecz, Ivetta Siket Mányiné, Lilla Mártha, Gábor Áron Fülöp, Tamás Radovits, Miklós Pólos, Béla Merkely, Árpád Kovács, Zoltán Szilvássy, Zoltán Ungvári, István Édes, Zoltán Csanádi, Judit Boczán, István Takács, Gábor Szabó, József Balla, György Balla, Petar Seferovic, Zoltán Papp, Attila Tóth

**Affiliations:** 1 Division of Clinical Physiology, Department of Cardiology, Faculty of Medicine, University of Debrecen, 22 Móricz Zsigmond street, Debrecen, 4032 Hungary; 2 Department of Cardiology, Faculty of Medicine, University of Debrecen, Debrecen, Hungary; 3 Doctoral School of Kálmán Laki, University of Debrecen, Debrecen, Hungary; 4 Department of Cardiac Surgery, University of Halle, Halle (Saale), Germany; 5 Department of Surgery, Faculty of Medicine, University of Debrecen, Debrecen, Hungary; 6 Heart and Vascular Center, Semmelweis University, Budapest, Hungary; 7 Department of Pharmacology and Pharmacotherapy, Faculty of Medicine, University of Debrecen, Debrecen, Hungary; 8 Vascular Cognitive Impairment and Neurodegeneration Program, Oklahoma Center for Geroscience, University of Oklahoma Health Sciences Center, Oklahoma City, OK USA; 9 International Training Program in Geroscience, Doctoral School of Basic and Translational Medicine/Department of Public Health, Semmelweis University, Budapest, Hungary; 10 Department of Neurology, Faculty of Medicine, University of Debrecen, Debrecen, Hungary; 11 Division of Nephrology, Department of Internal Medicine, Faculty of Medicine, University of Debrecen, Debrecen, Hungary; 12 HAS-UD Vascular Biology and Myocardial Pathophysiology Research Group, Hungarian Academy of Sciences, Budapest, Hungary; 13 Department of Pediatrics, Faculty of Medicine, University of Debrecen, Debrecen, Hungary; 14 Heart Failure Center, Faculty of Medicine, University of Belgrade, Belgrade, Serbia

**Keywords:** Angiotensin converting enzyme 2 (ACE2), Cardiovascular disease, Hypertension, Heart failure, Renin-angiotensin-aldosterone system (RAAS), RAAS inhibitors, SARS-CoV-2

## Abstract

Angiotensin-converting enzyme 2 (ACE2) is essential for SARS-CoV-2 cellular entry. Here we studied the effects of common comorbidities in severe COVID-19 on ACE2 expression. ACE2 levels (by enzyme activity and ELISA measurements) were determined in human serum, heart and lung samples from patients with hypertension (*n* = 540), heart transplantation (289) and thoracic surgery (*n* = 49). Healthy individuals (*n* = 46) represented the controls. Serum ACE2 activity was increased in hypertensive subjects (132%) and substantially elevated in end-stage heart failure patients (689%) and showed a strong negative correlation with the left ventricular ejection fraction. Serum ACE2 activity was higher in male (147%), overweight (122%), obese (126%) and elderly (115%) hypertensive patients. Primary lung cancer resulted in higher circulating ACE2 activity, without affecting ACE2 levels in the surrounding lung tissue. Male sex resulted in elevated serum ACE2 activities in patients with heart transplantation or thoracic surgery (146% and 150%, respectively). Left ventricular (tissular) ACE2 activity was unaffected by sex and was lower in overweight (67%), obese (62%) and older (73%) patients with end-stage heart failure. There was no correlation between serum and tissular (left ventricular or lung) ACE2 activities. Neither serum nor tissue (left ventricle or lung) ACE2 levels were affected by RAS inhibitory medications. Abandoning of ACEi treatment (non-compliance) resulted in elevated blood pressure without effects on circulating ACE2 activities. ACE2 levels associate with the severity of cardiovascular diseases, suggestive for a role of ACE2 in the pathomechanisms of cardiovascular diseases and providing a potential explanation for the higher mortality of COVID-19 among cardiovascular patients. Abandoning RAS inhibitory medication worsens the cardiovascular status without affecting circulating or tissue ACE2 levels.

## Introduction

The coronavirus disease 2019 (COVID-19) pandemic, caused by severe acute respiratory syndrome coronavirus-2 (SARS-CoV-2) resulted in a global health crisis. As of May 7, 2021, there were 156,465,211 cases and 3,264,509 victims of COVID-19 registered worldwide [1]. SARS-CoV-2 is highly virulent, with high mortality rates among hypertensive patients [2–4]. The mechanisms by which hypertension promotes increased COVID-19 mortality are complex and likely involve the interaction between hypertension, ageing and underlying cardiovascular comorbidities [5–7].

Cellular entry of the SARS-CoV-2 requires the binding of its spike protein to angiotensin-converting enzyme 2 (ACE2) on the cell surface [8]. There are speculations that modulation of this interaction directly (e.g. by blocking the binding) or indirectly (e.g. by trapping the coronavirus by soluble ACE2-like receptors) may provide a treatment option in COVID-19 [8–12]. Evolution of SARS-CoV-2 also targets the ACE2 binding site, as mutations in the ACE2 binding (spike) region of SARS-CoV-2 increased its virulence dramatically, resulting in the third wave of the pandemic [13]. Recent studies demonstrate that seriously ill COVID-19 patients exhibit significant increases in circulating ACE2 activity, suggesting that circulating ACE2 may predict disease severity [14, 15].

ACE2 is a component of the renin-angiotensin-aldosterone system (RAAS) and its expression is dysregulated in a range of cardiovascular diseases [16]. As ACE2 serves as cellular receptor for SARS-CoV-2, it has been proposed that its upregulation may contribute to the increased COVID-19 mortality in patients with cardiovascular comorbidities. Circulating ACE2 is a useful biomarker of changes in RAAS associated with different pathologies. Yet, changes in circulating ACE2 is patients at-risk for COVID-19 mortality have not been well documented.

A recent scientific discussion, based on indirect evidence from preclinical studies as well as from initial clinical observations, raised the possibility that inhibitors of the renin angiotensin aldosterone system (RAASi) may worsen the outcome of coronavirus infections [2, 3, 17–20]. This supposition relies largely on results from animal models showing that angiotensin II type 1 receptor blockers (ARBs) elevate circulating ACE2 levels [21, 22]. These preclinical results were extrapolated to humans and lead to the postulation for an adverse effect of RAAS inhibitors during SARS-CoV-2 infections [2, 17–19]. However, the actual effects of RAASi on circulating ACE2 in humans are not well understood. Due to the lack of definite data, leading medical organisations released public statements supporting continuing medication with RAAS inhibitors and stressed the urgent need for additional clinical data to elucidate the links among cardiovascular diseases, RAASi medications and ACE2 expression as they relate to COVID-19 mortality risk [23–25].

The present study was conducted to provide a thorough evaluation of ACE2 levels and expression in human sera and tissue samples (heart and lung) in relation to risk factors for COVID-19 mortality, including cardiovascular diseases (hypertension, heart failure), advanced age, obesity and male sex [26]. We also addressed the effects of cardiovascular disease severity and RAASi medications on ACE2 levels. Nonetheless, we should also highlight that the performed study is a retrospective analysis; therefore, a properly powered prospective study needs to be performed to confirm ACE2 as a selection marker in COVID-19.

## Methods

### Subjects

Individuals were enrolled at the Faculty of Medicine, University of Debrecen, Debrecen, Hungary (healthy, hypertensive and pulmonary disease patients). The study was authorised by the Medical Research Council of Hungary (20753-7/2018/EÜIG). Healthy individuals were without cardiovascular treatments or maladies. Hypertensive patients were enrolled at regular outpatient visits in a consecutive manner. Patients with lung disease were enrolled before chest surgery, representing a mixed cohort of diseases, irrespectively to the patient’s cardiovascular status. Explanted heart (and blood) samples were collected upon heart transplantation at the Semmelweis University Heart and Vascular Center, Budapest, Hungary. The sample collection was authorised by the Medical Research Council of Hungary (ETT TUKEB 7891/2012/EKU (119/PI/12.)). The clinical characteristics of the subjects involved are summarised in Table 1.

**Table 1:** General characteristics of the enrolled individuals

		Healthy	Hypertensive patients			Patients with pulmonary disease			End-stage heart failure patients			
Number of enrolled individuals (*n*)		46	386	71	43	24	7	18	200	54	23	19
Gender (male/female; *n*)		21/25	230/156	39/32	18/25	19/5	5/2	14/4	157/43	40/14	15/8	16/3
Age (years, mean ± SD)		30 ± 9	62 ± 10	60 ± 9	59 ± 14	67 ± 8	63 ± 6	55 ± 16	52 ± 10	56 ± 8	51 ± 12	53 ± 12
Body weight (kg, mean ± SD)		78 ± 6	83 ± 17	89 ± 17	78 ± 14	78 ± 13	80 ± 10	70 ± 17	79 ± 14	81 ± 14	78 ± 16	80 ± 17
Body height (m, mean ± SD)		1.8 ± 0.03	1.68 ± 0.09	1.68 ± 0.08	1.68 ± 0.08	1.69 ± 0.07	1.70 ± 0.07	1.72 ± 0.08	1.74 ± 0.09	1.74 ± 0.09	1.73 ± 0.09	1.74 ± 0.09
BMI (kg/m^2^, mean ± SD)		25 ± 4	29 ± 5	31 ± 5	28 ± 4	27 ± 5	28 ± 4	23 ± 5	26 ± 5	27 ± 5	26 ± 5	26 ± 5
Current smoker (%)		ND	16	10	14	21	29	44	ND	ND	ND	ND
Diabetes (NIDDM/IDDM)		0/0	18/5	28/10	14/2	4/2	0/1	0/0	3/47	1/10	0/2	1/3
Ejection fraction (%, mean ± SD)		62 ± 2	54 ± 8	56 ± 8	59 ± 7	60 ± 8	62 ± 4	61 ± 6	23 ± 8	23 ± 6	23 ± 11	22 ± 11
Heart rate (beats/min, mean ± SD)		79 ± 11	73 ± 12	72 ± 13	77 ± 11	73 ± 12	78 ± 12	79 ± 14	78 ± 15	74 ± 13	82 ± 16	82 ± 14
Systolic blood pressure (mmHg, mean ± SD)		123 ± 11	134 ± 19	134 ± 18	128 ± 16	134 ± 15	137 ± 15	133 ± 21	NA	NA	NA	NA
Diastolic blood pressure (mmHg, mean ± SD)		80 ± 10	80 ± 13	81 ± 10	80 ± 9	80 ± 9	83 ± 10	82 ± 8	NA	NA	NA	NA
Antihypertensive medication	Diuretics (%)	0	55	60	5	54	0	0	94	96	100	47
	*β-*blocker (%)	0	84	79	79	83	43	11	67	68	39	16
	αl-inhibitor (%)	0	7	7	5	8	0	0	0	0	4	0
	ACE-inhibitor (%)	0	100	0	0	100	0	0	100	0	0	0
	ARB (%)	0	1	100	0	0	100	0	2	100	0	0
	Ca-channel blocker (%)	0	37	48	21	46	14	11	3	4	0	0
	Imidazoline agonist	0	2	6	0	0	0	0	0	0	0	0

### Sample collection

Blood samples were collected in BD Vacutainer tubes (SST II Advanced, Ref. No. 367955). Samples were kept at room temperature until coagulation completed (at least 20 min), then serum was separated by centrifugation (1500 *g*, 15 min) and the supernatant (blood serum) was stored at −80 °C until the measurements. Blood sampling was performed according to the general aseptic technique upon regular visits (hypertensive patients); at the time of medical examination (healthy individuals); immediately before chest surgery (pulmonary diseases) and at a regular visit before heart transplantation. Lung tissue samples were biopsied from resected lung tissue without apparent (macroscopic) signs of tumorous infiltration. Lung tissue samples were collected from patients with adenocarcinoma (*n* = 24), squamous cell carcinoma (*n* = 9), metastatic tumours in the lung (*n* = 7), emphysema (*n* = 3), pneumonia (*n* = 2), sarcoidosis (*n* = 1), pneumothorax (*n* = 1), fibroelastosis (*n* = 1) and silicoanthrocosis (*n* = 1). Lung tissue samples were placed in a physiological buffer solution in the operating theatre and transferred to the laboratory. Here biopsies were made and the samples were frozen in liquid nitrogen and stored at −70 °C. Well-characterised pseudonymised blood serum and human myocardial tissue samples were obtained from the Transplantation Biobank of the Heart and Vascular Center at Semmelweis University, Budapest, Hungary [27]. Blood samples were collected immediately before heart transplantation. Myocardial tissue samples were biopsied by a transmural cut from the anterior wall of the left ventricle of explanted hearts during heart transplantation, immediately frozen in liquid nitrogen and stored at −80 °C.

### Biochemical methods

#### ACE2 activity measurements from the sera

Measurements were performed as described earlier [28, 29]. The ACE2-specific fluorescent substrate (Mca-APK-DNP) was custom synthesised by Peptide2.com (purity >95%). Measured fluorescence intensities were calibrated using 7-Methoxycoumarin (Mca, Sigma, Cat. No. 235199) standard solutions. The fluorescent intensities (in arbitrary units, AU) were plotted as the function of the reaction time. The slope of the linear fit on the plotted data yielded the measure of the activity (in AU/min) and accepted only if the goodness of the fit (*r*^2^) was higher than 0.9. The actual amount of the converted substrate was calculated based on the calibration of the instrument with the known concentration (amount) of the fully cleaved substrate. In some cases, the nonspecific activity was also determined. Nonspecific activity (activity in the presence of 1.25 μM MLN-4760) was 6.3 ± 0.8% (mean ± SEM) of the activity in the absence of the ACE2 inhibitor in a set of 152 serum samples. This nonspecific activity was considered to be negligible (thus uncorrected activities are shown here). The unit (U) of activity is defined as micromoles/min. Note, MLN-4760 was applied at a supra-maximal concentration, since it inhibited the ACE2 activity in the low nanomolar range (IC_50_ values) in various models in our laboratory.

#### ACE2 activity and expression measurements from lung and heart tissue samples

Tissue samples were broken to small pieces (ranging 9–89 mg in weight) in a mortar with a pestle under liquid air. Three (frozen) pieces were selected from each disrupted tissue sample to yield triplicates in case of the lung, while single tissue pieces were used in the case of heart samples. Frozen tissue pieces were placed in a glass tissue grinder (homogeniser) and 10-μl/mg (or a minimum of 200 μl) homogenisation buffer (assay buffer supplemented with 0.6% Triton-X-100, kept on ice) was added. Tissue was completely homogenised by a rotating PTFE pestle on ice.

Tissue homogenates were centrifuged (16,000 *g* for 10 min at 4 °C) and supernatants were collected and kept on ice while protein concentration measurements by the BCA method. Aliquots from the samples were diluted to 0.5 mg/ml (lung) or 1 mg/ml(heart) and the protein content of the diluted samples was determined in parallel with the activity and concentration measurements.

ACE2 activities in the tissue homogenates were measured as described for the serum activity measurements. The differences are that 10-μl tissue extract was pipetted into the wells of the black microtiter plates, which was diluted by 90-μl homogenisation buffer. Then the assay was performed exactly as described for the serum ACE2 activity measurements (adding the same salt-substrate regent and performing the measurement in 200-μl final volume).

Note, the nonspecific activity was significantly higher in the tissue extracts than in serum samples, reaching 66 ± 2% and 11 ± 0.5% (lung and heart, respectively, mean ± SEM). Accordingly, the specific activities are shown in the case of tissue samples:

Specific activity = activity without ACE2 inhibitor – activity in the presence of ACE2 inhibitor

The unit (U) of activity is defined as micromoles/min.

Lung and heart tissue ACE2 expression was also determined by ELISA (Cat# DY933-05, R&D Systems, McKinley Place, MN, USA), according to the manufacturer’s instructions. The ACE2 concentration was measured in parallel with the activity measurements at 16-fold final dilutions of the original tissue supernatants. Tissue supernatants were kept on ice until the ELISA determinations and were processed on the same day as their preparation. The values were estimated using a standard set provided with the kit on each microtiter plate, and the values were normalised to the measured protein concentrations.

### Measurement of the biochemical efficacy of the ACE inhibitory treatment

Measurements were performed as described earlier [30–32] with modifications. Two aliquots were taken from the same serum sample. One aliquot was diluted twofold, while the other was diluted by 200-fold in ACE assay buffer. One hundred microliters of these differently diluted samples was added into the wells of a 96-well plate. The plate was warmed to 37 °C and then the pre-warmed substrate solution (100 μl, 1 mM N-[3-(2-furyl)acryloyl]-L-phenylalanylglycylglycine) was added to start the reaction. The level of inhibition was calculated according to the equation.

$$ \mathrm{Level}\ \mathrm{of}\ \mathrm{ACE}\ \mathrm{inhibition}=100-\left(\frac{\mathrm{serum}\ \mathrm{ACE}\ \mathrm{activity}\ \mathrm{at}\ 4-\mathrm{fold}\ \mathrm{dilution}}{\mathrm{serum}\ \mathrm{ACE}\ \mathrm{activity}\ \mathrm{at}\ 400-\mathrm{fold}\ \mathrm{dilution}}\ast 100\right) $$

For additional details on the calculation and the theoretical background, please refer to [32].

### Chemicals

All chemicals were from Sigma if not stated otherwise.

### Ethical approval

All studies were approved by the Regional and Institutional Ethics Committee, University of Debrecen (UD REC/IEC number: 3261-2010 for hypertensive patients and UDCC REC/IEC number: 4375-2015 for tissue samples) and by the Medical Research Council of Hungary. The pseudonymised blood serum and human myocardial tissue samples were obtained from the Transplantation Biobank of the Heart and Vascular Center at Semmelweis University, Budapest, Hungary. Following institutional and national ethical committee approval (ethical permission numbers: ETT TUKEB 7891/2012/EKU (119/PI/12.) and informed consent from patients. Pseudonymised clinical data were obtained from the database of the Transplantation Biobank. The research was in accordance with the tenets of the Helsinki Declaration.

### Statistical analysis

Nonparametric tests were applied. Mann-Whitney test was used when two groups were compared. Kruskal-Wallis test with Dunn’s multiple comparisons test was performed for multiple groups. Correlations were evaluated by the Spearman’s rank correlation test or by linear fit. Statistical analysis was made by Prism for Mac OS X (GraphPad Software, San Diego, CA, USA). Differences between the compared groups were considered to be significant when *P* value was lower than 0.05 (Mann-Whitney test) or the mean rank differences reached significance as indicated by the statistical software.

## Results

### ACE2 activity correlates with the stage of cardiovascular disease and being modulated by comorbidities

**Fig. 1:**
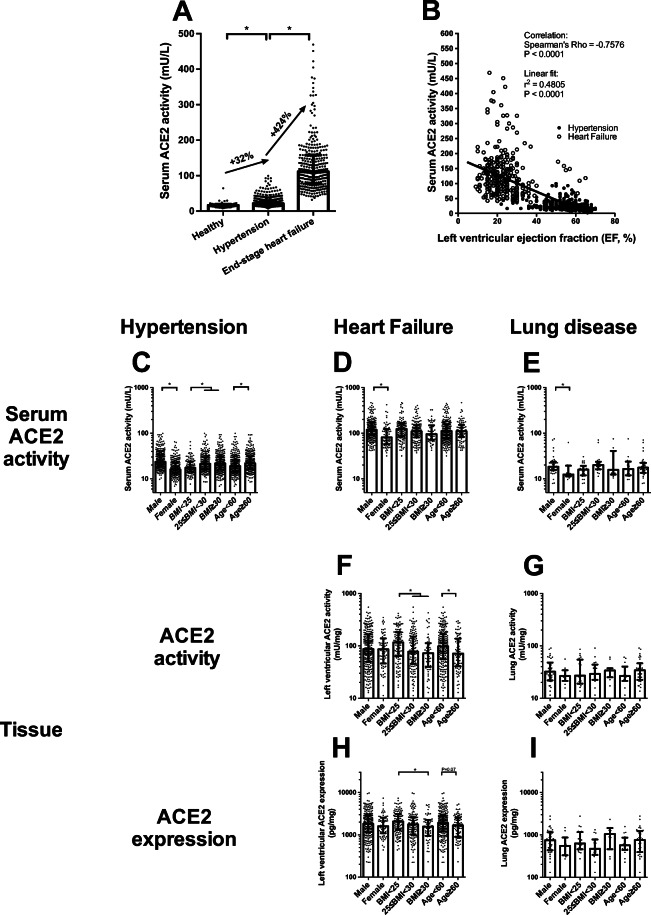
Serum and tissue ACE2 activities: a role for cardiovascular disease and comorbidities. Patients with different representation of the cardiovascular disease (healthy, hypertensive and end-stage heart failure) were grouped and the circulating ACE2 activity was measured. Patient groups are labelled below the bars. The circulating ACE2 activity (panels **A**, **B**, **C,D and E**), tissular ACE2 activity (panels **F** and **G**) and ACE2 tissular expression (panels **H** and **I**) were compared in groups representing cardiovascular comorbidities, including overweight, sex and age (labelled below the bars). Distribution of measured values is shown in scatter graphs (bars are median, error bars are IQR and symbols represent the individual patient’s data). Significant differences between the groups are represented by the brackets and asterisks

Circulating ACE2 activity correlated with the severity of cardiovascular disease. It was slightly elevated in hypertension (32% increase) and dramatically increased at the occurrence of heart failure (an additional 434% increase, Fig. 1A). Moreover, circulating ACE2 activity strongly correlated with the left ventricular ejection fraction (EF, Spearman’s rho = −0.7576, *P* < 0.05, *n* = 750, Fig. 1B), suggesting an almost linear correlation (parameters of the best linear fit on the values: *r*^2^ = 0.4805, *P* < 0.05, *n* = 750, Fig. 1B). Note, these data included all patients from the groups without selection by their medications (Table 2).

**Table 2:** Comparison of circulating and tissular ACE2 levels in different stages of cardiovascular disease

	Healthy (serum)	Hypertensive patients (serum)			End-stage heart failure patients (serum and heart tissue)			Patients with pulmonary disease (serum and lung tissue)	
	Without RAASi	Without RAASi	With ACEi	With ARB	Without RAASi	With ACEi	With ARB	Without RAASi	With RAASi
Number of enrolled individuals (*n*)	46	43	386	71	42	197	53	31	18
Serum ACE2 activity (mU/L, median ± IQR)	16 ± 7	17 ± 11	25 ± 15	21 ± 14	115 ± 73	112 ± 84	102 ± 81	18 ± 9	17 ± 10
Taking the ACEi drug (>90% inhibition)			26 ± 14						
Not taking the ACEi drug (<90% inhibition)			24 ± 16						
Tissular ACE2 activity (mU/mg, median ± IQR)	N/A	N/A	N/A	N/A	113 ± 120	115 ± 116	101 ± 104	32 ± 25	28 ± 23
Tissular ACE2 expression (pg/mg, median ± IQR)	N/A	N/A	N/A	N/A	1661 ± 1565	1900 ± 1694	1761 ± 2104	743 ± 950	515 ± 644

It was observed that the increase in circulating ACE2 activity is affected by cardiovascular comorbidities. Circulating ACE2 activity was higher in males than in females, and was elevated in overweight and obese patients; moreover, circulating ACE2 activity was also increased in elderly hypertensive individuals (Fig. 1C, Table 3).

**Table 3:** Confounding factors determining circulating and tissular ACE2 levels

		Gender		Obesity			Age < 60	
		Male	Female	BMI < 25	25 < BMI < 30	BMI > 30	Age < 60	Age > 60
Hypertensive patients	Number of individual samples	304	236	102	206	232	262	278
(Serum ACE2 activity, mU/L)	Median ± IQR	24 ± 15	16 ± 9	17 ± 9	21 ± 14	22 ± 15	19 ± 11	22 ± 15
End-stage heart failure patients	Number of individual samples	224	66	108	126	55	208	81
(Serum ACE2 activity, mU/L)	Median ± IQR	119 ± 80	81 ± 66	124 ± 84	110 ± 70	96 ±77	112 ± 80	111 ± 70
End-stage heart failure patients	Number of individual samples	226	66	112	124	56	213	79
(Left ventricular ACE2 activity, mU/L)	Median ± IQR	87 ± 115	86 ± 92	116 ± 120	77 ± 104	72 ± 73	98 ± 112	72 ± 97
End-stage heart failure patients	Number of individual samples	225	66	110	124	57	212	79
(Left ventricular ACE2 expression, pg/mg)	Median ± IQR	1818 ± 1700	1597 ± 1446	2067 ± 1798	1761 ± 1566	1564 ± 1297	1895 ± 1666	1669 ± 11,652
Patients with lung surgery	Number of individual samples	38	11	21	17	11	16	33
(Serum ACE2 activity, mU/L)	Median ± IQR	18 ± 8	12 ± 8	16 ± 7	20 ± 8	16 ± 28	16 ± 12	18 ± 9
Patients with lung surgery	Number of individual samples	37	11	20	20	11	15	33
(Lung ACE2 activity, mU/L)	Median ± IQR	32 ± 26	27 ± 13	27 ± 36	29 ± 21	34 ± 12	27 ± 19	34 ± 24
Patients with lung surgery	Number of individual samples	38	11	21	17	11	16	33
(Lung ACE2 expression, pg/mg)	Median ± IQR	750 ± 725	552 ± 536	627 ± 706	469 ± 443	1053 ± 994	568 ± 422	758 ± 844

A different effect of comorbidities was seen in end-stage heart failure patients. Among these patients, only the male sex resulted in significant differences in the circulating ACE2 activity (Fig. 1D, Table 3). In contrast, there was no effect of sex on the left ventricular ACE2 activity (Fig. 1F, Table 3) and expression (Fig. 1H, Table 3). However, both ACE2 activity and expression were lower in obese heart failure patients (Fig. 1F and H, Table 3). Similarly, ageing resulted in lower left ventricular ACE2 activity (Fig. 1F, Table 3).

Only male sex resulted in significant effects on the circulating ACE2 activities in the patient group with pulmonary disease (Fig. 1E, Table 3). There were no additional statistically significant differences in the circulating and tissue ACE2 levels (Fig. 1G and 1I, Table 3) in this group.

### Left ventricular ACE2 activity and expression correlate with circulating ACE2 activity

**Fig. 2:**
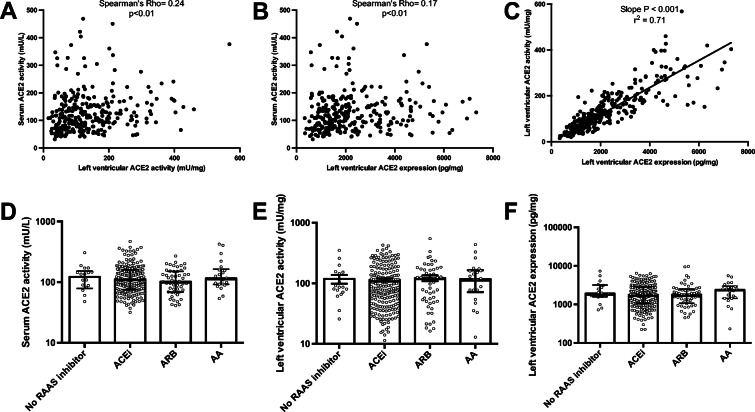
Serum ACE2 activity correlates with left ventricular ACE2 activity and expression. Serum ACE2 activity values were plotted as a function of left ventricular ACE2 activity (panel **A**) or left ventricular ACE2 expression (panel **B**). The correlation was tested by Spearman’s rank correlation and the main statistical parameters are shown in the inserts. Left ventricular ACE2 activity was also plotted as the function of left ventricular ACE2 expression (panel **C**). A linear correlation was established, and the fitted line is shown in the graphs. Each patient is represented by a single symbol on the graphs. The missing effects of RAASi medications on serum ACE2 activity (panel **D**), left ventricular ACE2 activity (panel **E**) and left ventricular ACE2 expression (panel **F**) are shown on scatter graphs (symbols are individual data, bars are median and error bars are IQR)

Both left ventricular ACE2 activity (Fig. 2A) and expression (Fig. 2B) positively correlated with the serum ACE2 activities of the same patients. Besides to these correlations, a linear relationship was verified between left ventricular ACE2 activity and expression (Fig. 2C), confirming that the two techniques are accurately measuring tissular ACE2 levels. Of note, there were no effects of RAAS inhibitory drug treatments on circulating ACE2 activities (Fig. 2D, Table 2), left ventricular ACE2 activity (Fig. 2E, Table 2) or left ventricular ACE2 expression (Fig. 1F, Table 2) in end-stage heart failure patients.

### Lung tissue ACE2 activity and expression did not correlate with circulating ACE2 activity

**Fig. 3:**
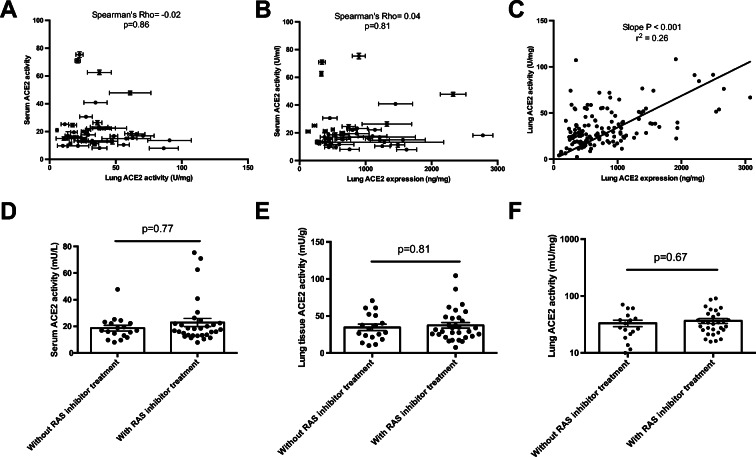
Missing correlation between serum ACE2 activity and lung ACE2 activity or lung ACE2 expression. Serum ACE2 activity values were plotted as a function of lung tissue ACE2 activity (panel **A**) or lung tissue ACE2 expression (panel **B**). No correlation was found by Spearman’s rank correlation (major statistical values are shown in the inserts). Lung tissue ACE2 activity was also plotted as a function of lung tissue ACE2 expression (panel **C**). A linear correlation was established, with the fitted line shown. All symbols represent an individual patient’s value. The missing effects of RAASi medications on serum ACE2 activity (panel **D**), lung tissue ACE2 activity (panel **E**) and lung tissue ACE2 expression (panel **F**) are shown on scatter graphs (symbols are individual data, bars are median and error bars are IQR)

Neither lung tissue ACE2 activity (Fig. 3A) nor lung tissue ACE2 expression (Fig. 3B) correlated with circulating ACE2 activities in the same patients with pulmonary disease. In contrast, a linear correlation was established between lung tissular ACE2 activity and expression (Fig. 3C). Similar to heart failure patients, RAAS inhibitory medication was without statistically significant effects on serum ACE2 activity (Fig. 3D, Table 2), lung tissular ACE2 activity (Fig. 3E, Table 2) and ACE2 expression (Fig. 3F, Table 2) in patients with lung disease.

### Circulating ACE2 was elevated in patients with primary lung cancer

**Fig. 4:**
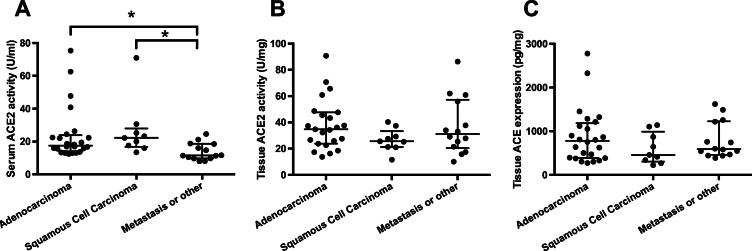
Serum ACE2 activity is elevated in patients with lung cancer. Serum ACE2 activity values were plotted in patients with pulmonary adenocarcinoma or squamous cell carcinoma or with metastasis in the lungs or other non-cancerous pulmonary malignancy (e.g. sarcoidosis, pneumonia, emphysema) on panel **A**. The dissected, macroscopically healthy surrounding tissue was also processed to measure ACE2 activity (panel **B**) and expression (panel **C**). Values are plotted on scatter graphs (symbols are individual data, bars are median and error bars are IQR). Statistical difference between the groups is labelled by the brackets and asterisks

Circulating ACE2 activity was elevated in patients with primary lung cancer (adenocarcinoma and squamous cell carcinoma) when compared to patients with metastasis or non-cancerous diseases (sarcoidosis, silicoanthracosis, pneumothorax, pneumonia, emphysema, Fig. 4A). In contrast, there was no difference in tissue ACE2 activity (Fig. 4B) or tissue ACE2 expression (Fig. 4C) in dissected, macroscopically healthy lung tissue samples.

### RAAS inhibitory medication is without effects on the circulating ACE2 activity in hypertensive patients

**Fig. 5:**
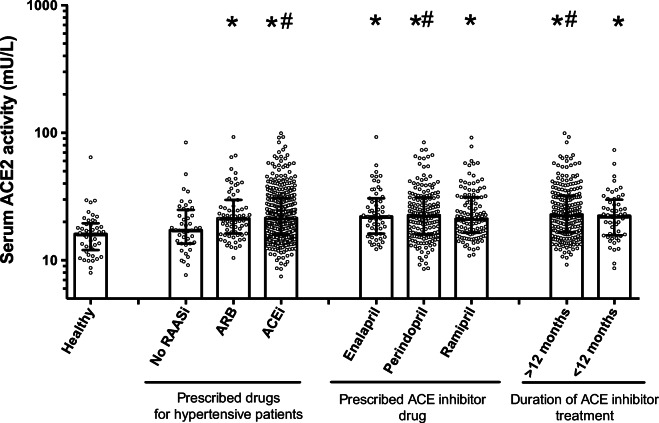
RAASi treatment does not affect serum ACE2 activity in hypertensive patients. Descriptive statistics are summarised in Tables 2 and 3, except for specific ACEi drugs: enalapril (*n* = 59), perindopril (*n* = 167) and ramipril (*n* = 113) and the durations for ACEi drug prescriptions: *n* = 223 patients had ACEi prescription for more than 12 months, and *n* = 52 for less than 12 months. Asterisks represent significant differences vs. the healthy group, while hashes indicate significant differences vs. the hypertensive patient group without RAASi medication. Each symbol represents an individual patient’s value

Serum ACE2 activities in hypertensive patients with ACEi drugs or with ARBs were higher, while ACE2 activities in hypertensive patients without RAAS inhibition overlapped with healthy controls (Fig. 5 and Table 2). Patients on different ACEi drugs (enalapril, perindopril and ramipril) had similar serum ACE2 activities (enalapril: 22 ± 14, *n* = 59; perindopril: 23 ± 15, *n* = 167; ramipril: 21 ± 15 mU/L, *n* = 113, Fig. 5). Furthermore, serum ACE2 activities were similar in patients treated for more (23 ± 16 mU/L, *n* = 223) or less than 12 months (22 ± 14 mU/L, *n* = 52) by ACEi drugs (Fig. 5).

### Insufficient compliance results in higher blood pressure, but does not affect circulating ACE2 activity in ACEi-treated hypertensive patients

**Fig. 6:**
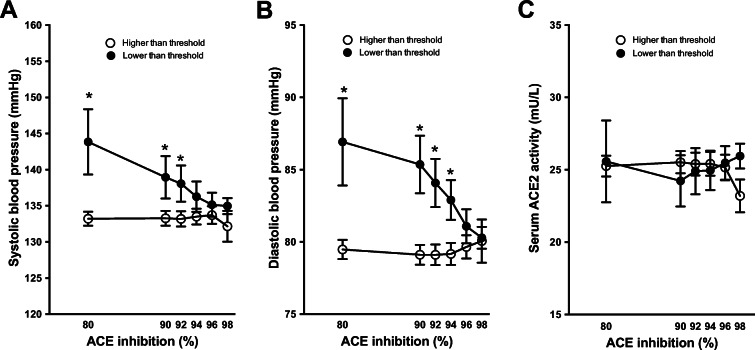
Insufficient compliance in ACEi treatment does not affect serum ACE2 activity, but increases blood pressure in hypertensive patients. Biochemical efficacy of ACEi medication was determined by a dilution-based biochemical test and threshold values were arbitrarily chosen. Mean systolic (panel **A**) and diastolic blood pressure (panel **B**) and serum ACE2 activity (panel **C**) are shown in patient’s population with lower than threshold (filled symbols) and higher than threshold (open symbols) biochemical ACE inhibition levels. Error bars are SEM. Significant differences (*p* < 0.05) among the groups (lower vs. higher ACE inhibition) are labelled by asterisks

Both systolic and diastolic blood pressure negatively correlated with the level of ACE inhibition in hypertensive patients on ACEi medication. The maximal anti-hypertensive effect of ACEi treatment on systolic (Fig. 6A) and diastolic (Fig. 6B) blood pressure was reached at 94 and 96% of ACE inhibition, respectively. In contrast, the level of ACE inhibition was without effects on serum ACE2 activity (Fig. 6C, Table 2).

## Discussion

The major findings of the present study are that expression of ACE2, the receptor for SARS-CoV [8, 33, 11], is modulated by a range of cardiovascular risk factors and pathologies, including hypertension, old age, comorbid obesity and heart failure.

We utilised a combination of reliable methods to assess circulating ACE2 as a potential predictive biomarker that could be used to stratify patients at risk for increased COVID-19 mortality. It is a strength of the current investigation that we (1) provided the measured ACE2 parameters in SI units; (2) confirmed tissular ACE2 activity by ELISA and (3) showed that ACE2 levels and activity values linearly correlate. Moreover, the evaluation presented here makes it possible to directly compare hypertensive and end-stage heart failure patients with healthy individuals to shed light on the potential role for ACE2 in cardiovascular disease. It is important to note that some other studies [34, 35] used to estimate circulating ACE2 levels based on a multiplexed qPCR-based technique [36], which does not yield real concentration values.

The first mentioning of ACE2 as a potential contributor to hypertension came from animal models. It was suggested that the ACE2 locus was located in a quantitative trait locus for blood pressure and it was confirmed that ACE2 levels are higher in spontaneously hypertensive rats [37]. Moreover, ACE2 deletion results in (albeit strain dependent) blood pressure elevation [38]. The above links have been subsequently extended to humans by our previous observations and thus implicating a role for ACE2 dysregulation in essential hypertension [28]. Here we put ACE2 in the context of cardiovascular disease continuum. Accordingly, the elevation in circulating ACE2 levels is relatively small in hypertension, especially when compared to end-stage heart failure patients. Serum ACE2 levels in end-stage heart failure patients were about fourfold higher than those in hypertensive subjects and about twofold higher than those in heart failure patients of low NYHA classes [28, 29]. Our previous studies revealed an inverse relationship between serum ACE2 activity and left ventricular ejection fraction in heart failure patients with reduced ejection fraction (HFrEF) and in patients with hypertension [28, 29, 39]. This proposed negative correlation between circulating ACE2 activity and left ventricular ejection function has been confirmed here, in a large cardiovascular patient population ranging from mild hypertensives to end-stage heart failure patients. The observed correlation appears to be strong (as defined by the high correlation coefficient rho value) and also linear, as represented by the linear distribution of the values. On the other hand, our results are in clear contrast to those obtained in experimental studies in laboratory rodents [21, 22], whereby we found no effects of RAASi medications on ACE2 activities or expressions in sera, left ventricular or lung tissues in humans.

Here we found about 50% higher circulating ACE2 activities in hypertensive patients with RAAS inhibitory medications (ACEi and ARB) than those in healthy individuals. This finding is of importance, as previous studies suggested that RAASi medication may contribute to COVID-19 mortality [2, 17–19], a hypothesis based on (1) the relatively high incidence of hypertension among SARS-CoV-2-infected patients [2]; (2) the findings of preclinical investigation showing that some RAASi medications result in increases in circulating ACE2 levels in animal models [21, 22]; and (3) the observation that ACE2 is critical for the binding and entry of SARS-CoV-2 to the cells [8, 9]. In the present study, we investigated the effects of RAASi medications on ACE2. A novel method was employed to determine the efficacy levels of ACE inhibitory drugs [32], thus allowing testing the hypothetical relationship between circulating ACE2 activities and the efficacies of ACEi medications. This analysis confirmed that patients with insufficient compliance had higher blood pressure values. More importantly, however, the level of ACE inhibition was without influence on ACE2 activities in the circulation. Accordingly, discontinuation of ACEi medication should not substantially affect serum ACE2 levels.

Circulating ACE2 activities were generally higher in males than in females, confirming previous reports [35, 34]. Moreover, the fact that this effect of gender is present in all patient populations (hypertensive, end-stage heart failure and pulmonary disease) points towards a common, disease independent expression/secretion mechanism. We also investigated tissular ACE2 expression patterns. Surprisingly, in contrast to the uniformly higher circulating ACE2 levels in males, left ventricular and lung tissue ACE2 levels showed no sex differences. The biological mechanisms underlying these observations are presently unclear. Additional differences were also noted between hypertensive and end-stage heart failure patients. Higher body mass index was paralleled by increased serum ACE2 activities only in hypertensive patients. We also found an age-dependent increase in serum ACE2 activity in hypertensive patients, extending recent findings [34].

On the basis of our findings, the contribution of different organs to the circulating ACE2 pool can be deduced. We propose that circulating ACE2 activity is a good biomarker of cardiac ACE2 expression [40], whereas its link to ACE2 expression in the lung is limited. Previous preclinical findings support this concept [41]. As the heart can be directly infected by SARS-CoV-2, circulating ACE2 could be used as a biomarker to identify patients at risk for cardiac injury associated with COVID-19.

The apparent disconnect between lung and circulating ACE2 levels can be explained by the high ACE2 expression level in the heart (being about fourfold higher than that in the lung, see Table 2)and/or by a more efficient ACE2 shedding from the heart than from the lung. ADAM17 was proposed as an ACE2 sheddase [42], which was found to be regulated to contribute presynaptic neuron activity [43] and upregulated in diabetes [44]. These findings suggest that ACE2 shedding to the circulation is not only depend on its expression, but also on ADAM17 expression and activity in the tissues. This may explain why the richly endothelised lung contributes less to the circulating ACE2 pool, then the heart.

Furthermore, we found that circulating ACE2 levels were elevated in patients with primary lung tumours. This elevation of circulating ACE2 activity cannot be explained by higher ACE2 expression in lung regions without (macroscopic) tumorous infiltration, since tissue ACE2 activities were not affected in the surrounding regions. This is important information in light that elevated circulating ACE2 levels were linked to elevated lung ACE2 expression [45]. Our data support increased ACE2 expression and secretion directly from the tumorous tissue in accordance with Zhang et al. [45], or some compensatory ACE2 secretion from non-cancerous tissues in these patients. A prime candidate of that can be the heart, having elevated right ventricular (and left atrial) blood pressure as a result of elevated pulmonary blood pressure. Clarifying this issue requires additional studies.

It is also possible that organs other than the heart also contribute to circulating ACE2 levels. For example, a recent study showed that ACE2 is highly expressed in the gastrointestinal tract and in the kidney [46]. In that regard, it is interesting that ~70% of patients infected with SARS-CoV-2 have gastrointestinal symptoms [47, 48] and a significant percentage develops acute kidney disease [49, 50]. These facts rise the possibility that the elevated circulating ACE2 levels not only reflects to ACE2 dysregulation in the heart, but may also indicate dysregulation in the kidneys. In particular, kidney failure is a common comorbidity of heart failure, and acute kidney failure may significantly contribute to the mortality in COVID-19 [49, 50]. While increased tissue ACE2 levels likely facilitates infection of cells with SARS-CoV-2, the effect of elevated circulating levels of ACE2 per se on the pathogenesis of COVID-19 is less understood [51].

There are certain limitations of the present work. A prime limitation is that it represents a retrospective analysis of our previous clinical studies and collected tissue samples. The samples have been drawn from the ethnically homogeneous (Caucasian) population of Hungary and therefore our study design precluded the analysis of the effects of race. Another limitation is that biospecimens derived from SARS-CoV-2-infected individuals were unavailable to test the predictive effects of the parameters assessed. Our experiments were not designed nor powered sufficiently to the question of the risk-benefit of RAAS inhibitors in COVID-19. Finally, the sources of the lung tissues were from various baseline diseases (cancer, sarcoidosis and pneumothorax were the most frequent diagnoses in the related experimental group).

In spite of these limitations, our study suggests that ACE2 dysregulation associates with the severity of cardiovascular disease, which can be a pathological step in the worsening of the patient’s cardiovascular condition. This pathological pathway may be particularly important in COVID-19, since ACE2 dysregulation may explain the higher mortality among elderly and overweight cardiovascular patients, implicating circulating ACE2 as a biomarker of COVID-19 mortality.
